# Associations of Green Spaces and Streets in the Living Environment with Outdoor Activity, Media Use, Overweight/Obesity and Emotional Wellbeing in Children and Adolescents

**DOI:** 10.3390/ijerph17176321

**Published:** 2020-08-31

**Authors:** Tanja Poulain, Carolin Sobek, Juliane Ludwig, Ulrike Igel, Gesine Grande, Verena Ott, Wieland Kiess, Antje Körner, Mandy Vogel

**Affiliations:** 1 LIFE Leipzig Research Center for Civilization Diseases, Leipzig University, Philipp-Rosenthal-Strasse 27, 04103 Leipzig, Germany; csobek@life.uni-leipzig.de (C.S.); jludwig@life.uni-leipzig.de (J.L.); wieland.kiess@medizin.uni-leipzig.de (W.K.); antje.koerner@medizin.uni-leipzig.de (A.K.); mvogel@life.uni-leipzig.de (M.V.); 2 Department of Women and Child Health, University Hospital for Children and Adolescents and Center for Pediatric Research, Leipzig University, Liebigstrasse 20a, 04103 Leipzig, Germany; 3 Center for Research and Transfer (FTZ) at the Leipzig University of Applied Sciences (HTWK), Research Field Health and Social Affairs, P.O. Box 30 11 66, 04251 Leipzig, Germany; ulrike.igel@htwk-leipzig.de (U.I.); gesine.grande@htwk-leipzig.de (G.G.); 4 Leibniz Institute for Regional Geography (IfL), Research Group Mobilities and Migration, Schongauerstrasse 9, 04328 Leipzig, Germany; v_ott@leibniz-ifl.de

**Keywords:** urban living environment, outdoor activity, overweight, emotional wellbeing, children

## Abstract

Aspects of the living environment can affect health and wellbeing of children and adolescents. Whereas most previous studies assessed the more distant residential urban environment, less is known on possible effects of the close environment. The present study investigated associations of the proportion of streets and green spaces in the immediate urban living environment (50, 100 and 400 m around the home) with media use, outdoor activity, overweight/obesity and emotional problems in two samples of younger (age 3–10, *n* = 395) and older children (age 10–19, *n* = 405). Independently of socioeconomic parameters, a higher proportion of streets was associated with overweight/obesity (in younger and older children), higher media use (in younger children), less outdoor activity and more emotional problems (in older children). Older children’s outdoor activity in winter increased with increasing proportions of green spaces. The observations suggest that the immediate urban living environment is a factor that can affect leisure behavior and health in children.

## 1. Introduction

The present study investigated associations between the immediate living environment in a German city and different aspects of behavior and health in children and adolescents. Socioecological approaches such as the Ecological Framework for Human Development [1] underline the importance and interactions of individual prerequisites and the immediate as well as the more distant physical and social environment in shaping behavior, wellbeing and health. Residential environments, as part of this physical and social environment, differ in terms of the presence of green spaces, playgrounds, street network and traffic load. Children and adolescents are creative actors who shape their living environment and develop coping strategies to get the best out of any environment. Nevertheless, the immediate living environment may have a direct or indirect effect on leisure behavior and health, especially when mobility is restricted, e.g., in younger children. The potential effects of the built environment (as well as the availability of food stores and leisure facilities) on activity and weight is also emphasized by the obesogenic environment approach [2].

In the last decades, a growing body of research explored associations between properties of the living environment, especially the urban living environment, and leisure activities, cognitive functioning and health. Physical activity and outdoor play have health-promoting effects on physical (e.g., weight [3]) and mental health (e.g., depressive symptoms [4]) in children and adolescents. However, according to a Europe-wide survey, only 25% of boys and 15% of girls meet current WHO recommendations to be active for at least 60 min per day [5]. Previous studies conducted in the US, Australia or Europe suggest that the amount of physical activity of children growing up in urban areas depends at least partly on properties of their living environment. Higher proportions of green spaces [6,7,8,9,10,11,12,13], more playgrounds [7] and fewer or more attractive and safe streets [11,12] were shown to be associated with more physical activity and more time spent outdoors. The same aspects of the environment have been shown to affect children’s active transportation [14,15,16].

Whereas the links between environment and physical activity have been investigated in several studies, potential associations with sedentary behavior such as media use are not well explored. Electronic media use has gained popularity in the last decades, and excessive use has been linked to several physical and mental health concerns in children and adolescents, e.g., overweight or lower mental health [17,18]. Two studies including children from urban as well as rural areas growing up in Australia [19] or Scotland [20] showed negative associations between the availability of green spaces and the media use of (young) children and argue that this association might be explained by the mobility and outdoor activity of children. Studies investigating the link between environment and media use in older children and adolescents or looking at environmental parameters other than green spaces are still lacking.

Importantly, not only leisure behavior but also parameters of physical and mental health [21] have been linked to the urban living environment. Regarding BMI or overweight, a major health problem in childhood and adolescence [22,23,24], previous studies revealed negative associations with the proportion of green areas [7,10,25]. Regarding other aspects of the urban living environment, e.g., the amount or safety of streets, a prospective study conducted in urban Canada showed a significant association between the availability of pedestrian walks and a lower BMI [26]. This finding might suggest that an increased road safety (indicated by available pedestrian routes) can have a positive effect on weight, e.g., by promoting mobility on foot. This suggestion is supported by another study showing significant associations of a higher perceived traffic density and a lower perceived traffic safety with overweight/obesity in children and young adolescents in urban Australia [27].

With respect to the emotional wellbeing of children, previous studies showed negative associations between the quantity and quality of green spaces in the urban living environment and internalizing/emotional problems in children [28] and adolescents [13]. These studies suggest a positive effect of urban green spaces on children’s mood. However, in other studies, the proportion of green spaces was not related to emotional problems in children [29,30]. Regarding other parameters of the environment, previous investigations conducted in two German cities underline the potentially negative effects of road traffic noise on emotional wellbeing in children growing up in urban areas [31,32].

An important factor that has to be taken into account in studies on the living environment is the socioeconomic status (SES) of persons/families living in different residential areas. People from lower social strata are more likely to live in less attractive and less mobility-friendly residential environments, e.g., in environments with fewer green spaces [33,34]. At the same time, they show a less healthy behavior and more health problems [35]. It is, therefore, important to include individual or regional parameters of social deprivation as possible confounders in the assessment of associations between living environment and health.

Overall, several previous studies investigated the relationship between aspects of the urban living environment and single aspects of health and behavior. However, while the potential of green spaces has been studied intensively, less is known on possible effects of streets. A high proportion of streets in urban residential areas could pose a safety risk for outdoor activities and have a negative effect on wellbeing through increased noise levels. In addition to this, no study comprehensively explored associations of the urban living environment with active versus passive leisure behavior, and aspects of physical as well as mental health in the same study. Here, we investigated associations of the proportions of green spaces and streets in the immediate urban living environment with leisure activities (outdoor play and media use), overweight/obesity and emotional problems in a large sample of children and adolescents. Importantly, while most previous studies assessed these parameters of the living environment in a rather large radius around the home, here, we explicitly investigated the immediate living environment and differentiated among three radiuses (50, 100 and 400 m distance from home). Based on previous studies [6,7,8,9,10,11,12,13,19,20,21,25,28], we hypothesized that green spaces are associated with less media usage, more outdoor play, a lower proportion of overweight/obesity and a lower prevalence of emotional problems. Higher amounts of streets, in contrast, were hypothesized to go along with a higher media usage, less outdoor play, a higher frequency of overweight/obesity and a higher prevalence of emotional problems. Family SES and a marker of social deprivation in the residential district were included as covariates (in addition to gender and age). Furthermore, we explored whether the strengths of associations between the urban living environment and leisure activities/health differ depending on SES or gender.

## 2. Materials and Methods 

### 2.1. Participants

The project was realized between 2018 and 2019 in the framework of the LIFE Child study, a longitudinal study aiming to monitor the development of healthy children and adolescents, with a special focus on civilization diseases such as overweight and obesity [36,37]. Participants come from Leipzig, Germany, a city located in eastern Germany, with about 600,000 inhabitants and a surface area of about 30,000 ha [38]. In LIFE Child, participants are recruited between birth and 16 years of age, with subsequent annual follow-up visits. Children suffering from any chronic or syndromal diseases are excluded from the study. The study was designed in accordance with the Declaration of Helsinki and was approved by the Ethics Committee of the Medical Faculty of the University of Leipzig (Reg. No. 264/10-ek). Parents of all participants provided informed written consent. From the age of 12 years on, children themselves also provided informed written consent. The percentages of different land cover types were provided by the LIFE Child Research Center. According to the data protection law, no actual addresses were used for the current analyses.

For the present project, all children and adolescents with available information on BMI, leisure activities, emotional problems and parameters of their home environment were eligible. Based on child age and depending on who had completed the questionnaires on leisure activities and emotional problems (parents versus children themselves), two subsamples were distinguished: a sample of younger children (3–10-year-olds) in which parents had provided information and a sample of older children (10–19-year-olds) in which children themselves had provided information. In the case of multiple study visits per child, only the first visit was analyzed. Furthermore, to preserve statistical independence, only one child per family was included in the analyses. The final sample of younger children comprised 389 participants (mean age = 7.53, sd = 1.90, 213 (55%) boys). The final sample of older children comprised 403 participants (mean age = 13.50, sd = 2.51, 216 (54%) boys). For 84 of the older children, information on emotional problems was missing. These children were excluded from the analyses on emotional problems. The distribution of age (mean age = 13.23, sd = 2.03) and gender (54% male, 46% female) in this subsample was comparable to the distributions in the total sample of older children. 

### 2.2. Exposure Variables

Land-cover data for Leipzig were retrieved based on published data from the open-access library PANGAEA.de [39]. The authors used object-based image analysis on digital orthophotos taken 2012 with a ground resolution of 20 cm and derived altitude information to classify structures into different classes of buildings, places, streets and green spaces. The location of streets in Leipzig did not change since 2012. Regarding green spaces, there were only negligible changes.

Data were stored and processed in a PostGIS database. For different radiuses (50, 100 and 400 m), buffers around the home addresses of the participants were created and, subsequently, the percentages for the different land-cover types were calculated. The total percentages of green spaces (sum of agriculture, lawn, bushes/young trees and trees) and streets (sum of railways and streets) within 50, 100 and 400 m of the home were used for analyses in the present project.

### 2.3. Outcome Variables

Outdoor activity, media use and emotional difficulties were assessed via questionnaires completed by either participants themselves (sample of older children) or their parents (sample of younger children). 

Outdoor activity: The physical activity questionnaire was designed by the authors and adapted from the Active Where? Surveys [40]. Here, only the questions on the frequency of outdoor activities of at least one-hour duration were included. Separate questions were asked for outdoor activity during summer and winter. Five answer categories could be chosen: “less frequently than once a week”, “once a week”, “more frequently than once a week”, “on most (school) days” and “usually each day”. For further analyses, the frequencies of outdoor activities were dichotomized into “low/normal” or “high” (“usually each day” for younger children; “on most (school) days” and “usually each day” for older children) in such a way that a comparable number of participants was assigned to each category. 

Media use: An in-house designed media questionnaire was applied as previously described [41]. Participants were asked to indicate how much time per day they (or their children) usually spend using TV, games consoles, computers and mobile phones. Separate questions were asked for media use during the week or on weekends. For computers and mobile phones, online and offline use were distinguished. For each question, five response categories were provided: “never”, “30 min/day”, “1–2 h/day”, “3–4 h/day” and “more than 4 h/day”. In a next step, the responses were transformed into durations (“never” = 0, “30 min/day” = 0.5, “1–2 h/day” = 1.5, “3–4 h/day = 3.5, “>4 h/day” = 5), and the durations on weekends and weekdays were combined (duration on weekdays × 5 + duration on weekend × 2)/7). To estimate participants’ total daily media use, the durations of watching TV, using games consoles, computers (online + offline) and mobile phones (online + offline) were summed up. In a final step, total media use was categorized as either “low/normal” or “high” based on a median split. In the sample of younger children, all total media usage times above 1.28 h/day were considered as “high”. In the sample of older children, all total screen times above 5.0 were considered as “high”.

Overweight/obesity: Weight and height of study participants were measured by trained study assistants. The BMI was transformed into BMI standard deviation scores based on German age- and sex-adjusted references [42]. All standard deviation scores > 1.28 (>90th percentile) were categorized as overweight/obese [42].

Emotional problems: Emotional problems were assessed using the self- or parent-report version of the Strengths and Difficulties Questionnaire (SDQ), a well validated standard instrument on the behavioral difficulties of children and adolescents [43]. We included the scale “emotional problems” in the analyses, which contains five questions on the emotional wellbeing of children and adolescents, e.g., on feelings of sadness or loneliness. Each question can be answered on a three-point Likert scale (“not true”, “somewhat true” and “certainly true”). The final score ranges between 0 and 10, with higher scores indicating more emotional problems. The internal consistency (indicated by Cronbach’s alpha) was 0.65 in the sample of younger children and 0.69 in the sample of older children, which is comparable with the internal consistencies observed in German normative samples (0.66 [44] and 0.62 [45], respectively). Based on the German reference values, the score was dichotomized into “low/normal” (scores 0–3 in the sample of younger children [44] and scores 0–4 in the sample of older children [45]) and “borderline/abnormal” (all scores above 3 or 4, respectively). The cut-off values were chosen so that 15% of the children/adolescents were assigned to the “borderline/abnormal” group [44,45].

### 2.4. Covariates

Child age, child gender, family socioeconomic status (SES) and the rate of social benefit recipients (SBR rate) in the local district were included as covariates. For the assessment of family SES, parents provided information on their education, occupation and income (which was then transformed into the household equivalent income accounting for the number of persons living in the home). Information on these three parameters was combined in a SES composite score [46,47] ranging between 3 and 21, with higher scores indicating higher SES. The SBR rates per local district (*n* = 63) were provided by the municipality of the City of Leipzig and ranged between 2% and 39%.

### 2.5. Statistical Analysis

All statistical analyses were performed using R (R Foundation for Statistical Computing, Vienna, Austria) [48]. To assess associations of parameters of the urban living environment (as exposure variables) with outdoor activity, media use, overweight/obesity and emotional difficulties (as outcome variables), we applied logistic regression analyses. Separate models were calculated for each radius (50, 100 and 400 m) around the residential address. Child age, gender, family SES (composite score) and local SBR rate were included as covariates. To assess differences of associations depending on SES or gender, all models were checked for interactions between the exposure variables and family SES (composite score), local SBR rate or gender. An interaction was considered meaningful if it was significant (*p* < 0.05) and did not limit the quality of the statistical model (variance inflation factor (vif) < 5). In addition, urban district was added as a random effect to the model but did not explain any variance in the model. Therefore, it was removed to satisfy the principle of parsimony.

## 3. Results

### 3.1. Characteristics of the Study Population and Living Environment

A description of the study samples is shown in Table 1. The study population was shifted towards a higher SES, with almost half of the children in the samples of younger or older children (49% and 43%, respectively) belonging to the high SES group. The average SBR rates in the samples of younger and older children were 10.56 and 11.20, respectively. This is 3–4% lower than the average of the city of Leipzig (mean = 14.23). 

The proportion of streets ranged basically between 0% and 20%, whereas the amount of green spaces ranged mainly between 20% and 80%. Overweight/Obesity was present in 10% of the younger and nearly 20% of the older children. 

### 3.2. Associations between Parameters of the Environment and Leisure Activities and Health

**Figure 1:**
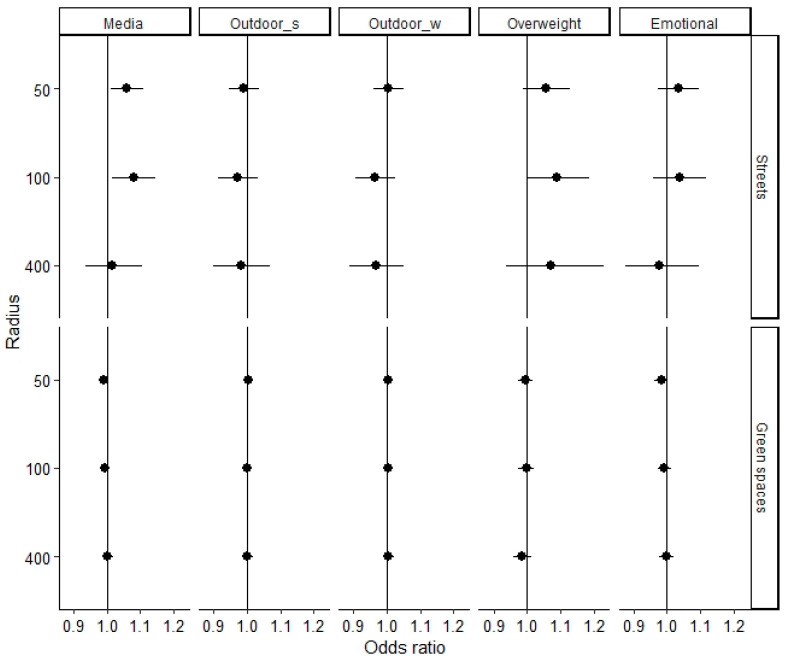
Associations (+95% CI) between parameters of the urban living environment and high media use (Media), frequent outdoor activity in summer (Outdoor_s) and winter (Outdoor_w), overweight/obesity (Overweight) and emotional problems (Emotional) in the sample of 3–10-year-old children. All associations are adjusted for child sex, age, family SES (composite score) and local SBR rate.

**Figure 2:**
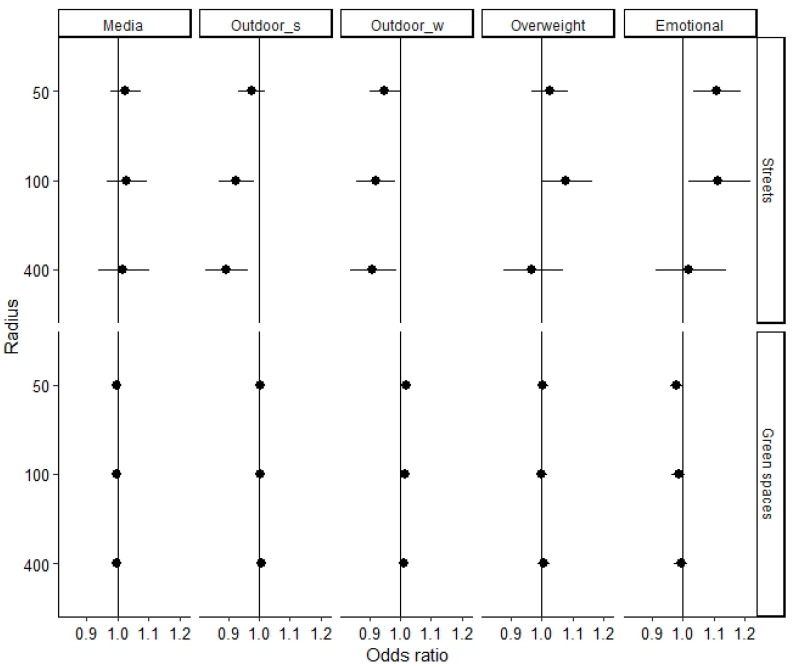
Associations (+95% CI) between parameters of the urban living environment and high media use (Media), frequent outdoor activity in summer (Outdoor_s) and winter (Outdoor_w), overweight/obesity (Overweight), and emotional problems (Emotional) in the sample of 10–19-year-old children. All associations are adjusted for child sex, age, family SES (composite score) and local SBR rate.

**Figure 3:**
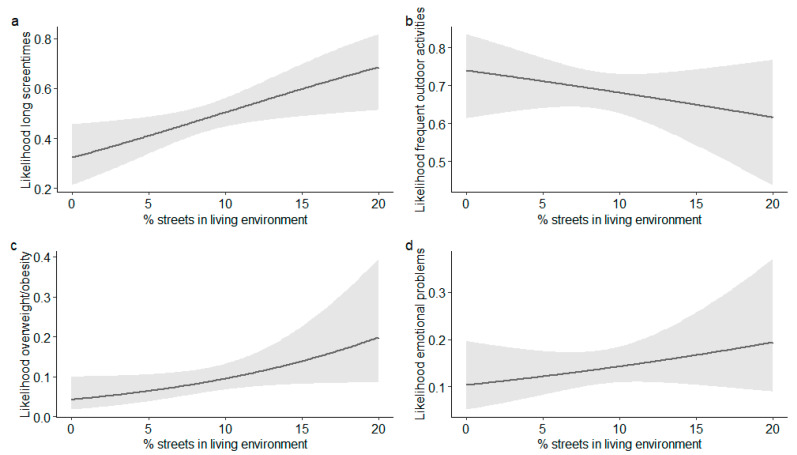
Effect plots illustrating the estimated effects (+95% CI) of the percentage of streets within 100 m of home on: (**a**) high screen times; (**b**) frequent outdoor activity in summer; (**c**) overweight/obesity; and (**d**) emotional problems in the sample of 3–10-year-old children.

The associations of the proportions of streets and green spaces within 50, 100 or 400 m of the home with outdoor activity, media use, overweight/obesity and emotional problems of the study participants are shown in Figure 1 (younger children) and Figure 2 (older children). In the sample of younger children, a higher percentage of streets within 50 and 100 m of the home was significantly associated with a high media usage (OR_50 m_ = 1.06 (95% CI 1.01–1.11)), OR_100 m_ = 1.08 (95% CI 1.02–1.15), see Figure 3). Furthermore, the analyses revealed a marginally significant positive association between the percentage of streets within 100 m and overweight/obesity (OR = 1.09 (95% CI 1.00–1.18), *p* = 0.052). For 5% and 20% of streets, the estimated likelihoods of overweight/obesity were 6% and 20%, respectively (see Figure 3). The analyses revealed no significant associations between parameters of the environment and outdoor activity or emotional problems in the younger children. Additionally, the percentage of green spaces was associated with none of the assessed outcome variables. 

**Figure 4:**
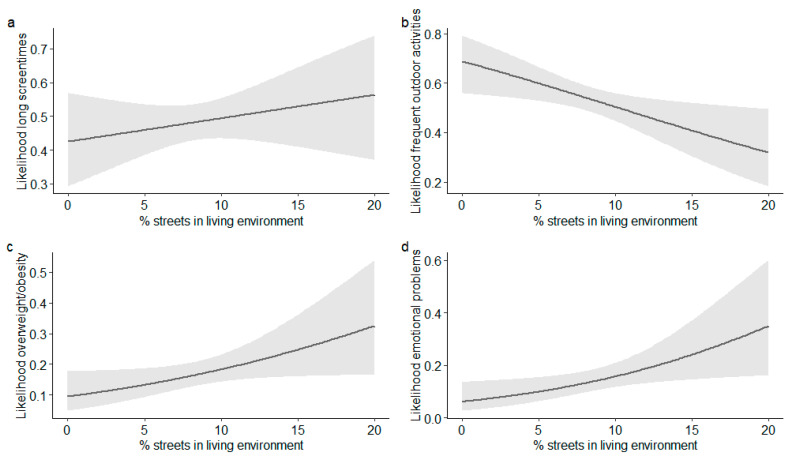
Effect plots illustrating the estimated effects (+95% CI) of the percentage of streets within 100 m of home on: (**a**) high screen times; (**b**) frequent outdoor activity in summer; (**c**) overweight/obesity; and (**d**) emotional problems in the sample of 10–19-year-old children.

In the sample of older children, a lower percentage of streets within 100 and 400 m of the home was significantly associated with an increased likelihood of outdoor activities in summer (OR_100 m_ = 0.93 (95% CI 0.87–0.98), OR_400 m_ = 0.89 (95% CI 0.83–0.97)) and winter (OR_100 m_ = 0.92 (95% CI 0.86–0.99), OR_400 m_ = 0.91 (95% CI 0.84–0.99)). The association between the proportion of streets within 100 m from home and outdoor activity in summer is displayed in Figure 4. Outdoor activity in winter (but not in summer) was also associated with a lower percentage of streets (OR = 0.95 (95% CI 0.90–0.99)) and a higher percentage of green spaces (OR = 1.02 (95% CI 1.00–1.04)) within 50 m of the home. For 20% of green spaces, the likelihood of frequent outdoor activity in winter was estimated at 20%. For 70% of green spaces, in contrast, the estimated likelihood was 40%. 

The analyses also revealed a significant positive association between a higher percentage of streets within 100 m of the home and the risk of overweight/obesity in adolescents (OR = 1.08 (95% CI 1.01–1.16)). For 5% and 20% of streets, the estimated likelihood of being overweight/obese was 13% and 32% (see Figure 4). Looking at emotional problems, a higher percentage of streets within 50 or 100 m of the home significantly increased the risk of borderline/high amounts of emotional problems (OR_50 m_ = 1.11 (95% CI 1.03–1.18)), OR_100 m_ = 1.11 (95% CI 1.02–1.22)). For 5% of streets in the buffer of 100 m from the home, the estimated likelihood of borderline/high amounts of emotional problems was 10%, compared to 35% in the case of 20% streets (see Figure 4). Media use of adolescents was associated with neither the percentage of streets nor the percentage of green spaces.

### 3.3. Differences in Strengths of Associations Depending on SES or Gender

All models were checked for interactions between the environmental exposure variables and the covariates family SES, local SBR rate and gender. Overall, seven interactions reached statistical significance (one for family SES, two for local SBR rate and four for gender). However, all of these interaction terms limited model quality by causing severe inflation of variance (all vif > 10) and, thus, were not included in the final statistical models. It can be considered that the strengths of associations between parameters of the environment and outdoor activity, media usage, overweight/obesity and emotional problems did not differ significantly depending on SES or gender.

## 4. Discussion

### 4.1. Summary of Main Findings

The present study investigated associations of characteristics of the immediate urban living environment (percentage of streets and green spaces within 50, 100 and 400 m from home) with leisure behaviors and parameters of physical and mental health in children and adolescents. 

In line with socioecological approaches, the proportion of streets was associated with the behaviors and health outcomes assessed in the present study (independently of family SES and rate of social welfare recipients). In younger children, we observed significant associations of the percentage of streets with media use and overweight/obesity. In older children, associations of the percentage of streets (and, partly, green spaces) with outdoor activity, overweight/obesity and emotional problems reached significance, indicating that environmental characteristics are associated with different parameters of health and behavior depending on the age of the child. Possible reasons for these discrepancies are differences in the perception and significance of living environments, as well as in the control and involvement of parents depending on child age. Even if the effect sizes were only moderate, the present findings underline the importance of the urban living environment in the context of child health. 

### 4.2. Associations between the Proportion of Streets and Leisure Behavior and Health

Based on the (limited) literature on the link between streets/traffic and behavior and health in children and adolescents, we expected significant associations of a higher percentage of streets in the urban living environment with less outdoor activity, more media use, a higher likelihood of overweight/obesity and more emotional problems. These hypotheses could largely be confirmed, with some differences according to age (child versus adolescent) or distance from home (50, 100 or 400 m). 

In line with our hypotheses, the analyses showed significant associations between a lower percentage of streets and a higher likelihood of regular outdoor activity in adolescents. This finding is in line with a previous study in adolescent girls from the cities of San Diego and Minneapolis in the US [12] and suggests that the proportion of streets in the urban living environment can influence whether children spend time outside or not. However, these associations were only significant in the sample of older children, suggesting that the importance of streets in the immediate urban environment only develops with increasing age, when children explore their environment themselves and are no longer dependent on their parents. At younger age, outdoor activity mainly depends on infrastructures in childcare facilities and on parents, who may accept longer walking or, if necessary, driving distances to allow their children to engage in outdoor activities. It is, therefore, possible that younger children who grow up in an urban environment with more streets also (as adolescents) spend less time in this environment, but that their outdoor activities are made possible in other places. 

Interestingly, we observed that a higher proportion of streets within the most immediate residential environment (buffer of 50 m from the home) was associated with less outdoor activity in winter. Outdoor activity in summer, in contrast, was not associated with the proportion of streets in the living environment. This finding may suggest that, in winter, when the motivation to spend time outdoors is limited due to unpleasant weather conditions, the most immediate environment in an urban area (e.g., the presence of traffic-calmed areas directly next to the house or apartment) is more relevant than in summer. 

While associations between streets and physical or outdoor activity have been investigated in several studies, the present study is, to the best of our knowledge, one of the first to explore associations between the proportions of streets and media use of children and adolescents. As expected, we observed a significant association between a higher proportion of streets and higher media use in younger children. One possible explanation for this association is that children growing up in a part of a city with more streets spend less time in their immediate environment, e.g., because of safety concerns, but more time with electronic media. The finding that this association could only be observed in the sample of younger children might be explained by differences in which and how media devices are used. Younger children mainly watch TV or use computers/tablets and are usually supervised by their parents at home. Adolescents, in contrast, mainly use mobile media devices such as smartphones, which are also used outside. 

In line with our hypothesis, we found that a higher percentage of streets in the urban living environment is associated with overweight/obesity in children. This finding is in line with previous studies showing associations between high traffic intensity or low traffic safety and more overweight/obesity in children from cities in Canada [26] and Australia [27]. The connection between the proportion of streets and overweight/obesity might be based on an association between urban living environment and passive or active leisure behavior. The observation that the association was only observable when considering the radius 100 m from home address might be further explored in future studies. 

Regarding the relationship between the proportion of streets and emotional wellbeing, we found that the likelihood of emotional problems in older children was significantly higher in parts of Leipzig with more streets. The proportion of streets in the most immediate environment (50 m from home) showed the strongest associations with emotional problems. Therefore, one could speculate that road traffic noise, streetlight or an unpleasant sight of streets are main explanatory factors in this context. However, these aspects of the environment were not investigated in the present study. Interestingly, the associations were only significant in the sample of older children, indicating that the potential negative effects of streets in urban living environments on emotional mood only develop or manifest with increasing age. Another potential explanation for the finding is that older children are more sensitive to or less resistant against the influences of the direct street environment (e.g., traffic noise, streetlight and unpleasant sight) than younger children. 

### 4.3. Associations between Green Spaces and Leisure Behavior and Health

Associations between the proportion or availability of green spaces in the living environment and leisure behavior or health have been observed in several studies [6,7,8,9,10,11,12,13,19,20,21,25,28]. Based on the results of these studies, we hypothesized that a higher proportion of urban green spaces is associated with more outdoor activities, less media use, a lower risk of overweight/obesity and fewer emotional problems in children and adolescents. Surprisingly, most of these hypotheses could not be confirmed. The only significant association observed in this study was a positive association between higher amounts of green spaces within 50 m of the home and more outdoor activity in winter (but not summer) in the sample of older children. This association suggests that green spaces in the most immediate urban living environment (e.g., presence of gardens or green space directly next to the house or apartment) plays a more essential role for outdoor activity in winter, when the motivation to go outside is lower than in summer. No other associations between the proportion of green spaces and leisure behavior or health could be found in this study. 

One possible reason for the discrepancies with previous studies are methodological differences. While the present study considered several rather small buffers around the residential address, other studies assessing urban living environments used larger buffers (e.g., 500 m [25] or 1000 m [8]) or information from local districts [7,10,19]. This might suggest that green spaces in the broader urban environment have a stronger impact on leisure behavior and health than green spaces in the immediate living environment (with the exception of the importance of green spaces for outdoor activity in winter). It is also important to note that different types or qualities of green spaces (e.g., fields, parks, playgrounds and small grassy areas next to buildings or roads) may have different effects on behavior and health. Unfortunately, it was not possible to differentiate between these types of green spaces in the data available here. This assumption might be investigated in studies including both very small and very large radiuses around the home address or in studies using different methodological designs, e.g., qualitative inquiries. 

### 4.4. Strengths and Limitations

The major strength of this study is the investigation of several parameters of urban environment, behavior, and health in large samples of 3–19-year-old children. One limitation is the over-representation of families from higher social strata. Therefore, generalizations to children growing up in lower social classes are limited. A further limitation is the concentration on urban living environments and, more specifically, on one city in Germany. Green spaces and streets in rural areas or in other regions of Germany or the world might show different associations with behavior and health (e.g., because streets might be quieter), e.g., due to differences in geography and urban planning. Regarding the leisure behavior of children and adolescents, it is important to note that we relied on reports of children or their parents, i.e., on subjective perceptions, which may over- or underestimate the real behavior. In addition, the categorizations into “normal” or “high” were based on observations made in this study rather than current recommendations. Therefore, “normal” screen times or “high” levels of outdoor activity cannot be equated with “healthy” amounts of media use or outdoor activity. 

## 5. Conclusions

The findings of the present study suggest that the structure of the immediate urban living environment (and especially the proportion of streets) can have an effect on active and passive leisure behavior as well as on BMI and emotional wellbeing, with some effects varying depending on child age. The results underline the importance of the living environment for behavior, health and wellbeing in childhood and adolescence and call for strategies to optimize urban environments. As it is difficult to change the amount of grey space and the number of streets in a city, urban planning might consider ways to reduce traffic and expand pedestrian zones.

## Figures and Tables

**Table 1:** Description of the study samples (younger children and older children).

	Younger Children (3–10)	Older Children (10–19)
**Demographics**		
*n*	389	403 ^a^
Male (*n*, %)	213 (55%)	216 (54%)
Female (*n*, %)	176 (45%)	187 (46%)
Age (mean, sd)	7.54 (1.90)	13.50 (2.51)
SES score (mean, sd)	16.13 (3.08)	15.45 (3.18)
Low SES (*n*, %) ^b^	6 (2%)	8 (2%)
Middle SES (*n*, %) ^b^	189 (49%)	223 (55%)
High SES (*n*, %) ^b^	194 (49%)	172 (43%)
Local SBR rate (mean, sd)	10.56 (6.77)	11.20 (7.92)
Percentage green spaces		
Within 50 m of home (mean, sd)	39.34 (15.68)	43.48 (15.38)
Within 100 m of home (mean, sd)	43.09 (14.95)	46.97 (15.31)
Within 400 m of home (mean, sd)	46.43 (13.94)	50.27 (15.00)
Percentage streets		
Within 50 m of home (mean, sd)	9.58 (4.73)	8.94 (4.52)
Within 100 m of home (mean, sd)	8.64 (3.65)	8.35 (3.40)
Within 400 m of home (mean, sd)	8.59 (2.74)	8.19 (2.88)
**Outdoor activity**		
Summer		
Low/normal (*n*, %)	127 (33%)	188 (47%)
High (*n*, %)	262 (67%)	215 (53%)
Winter		
Low/normal (*n*, %)	247 (63%)	285 (71%)
High (*n*, %)	142 (37%)	118 (29%)
**Media use**		
Total media use (in h (mean, sd))	1.69 (1.53)	5.73 (3.89)
Low/normal (*n*, %)	202 (52%)	209 (52%)
High (*n*, %)	187 (48%)	194 (48%)
**BMI/overweight/obesity**		
BMI (mean, sd)	−0.06 (0.97)	0.23 (1.20)
Normal weight (*n*, %)	349 (90%)	326 (81%)
Overweight/obesity (*n*, %)	40 (10%)	77 (19%)
**Emotional problems ^a^**		
SDQ score (mean, sd)	1.63 (1.73)	2.28 (2.10)
Low/normal (*n*, %)	332 (85%)	269 (84%)
Borderline/abnormal (*n*, %)	57 (15%)	50 (16%)

^a^ In the sample of older children, information on emotional problems was missing in *n* = 84 participants, resulting in *n* = 319 in this subsample. ^b^ Based on reference values achieved in a representative German sample [47], the SES score can be used to categorize the SES as low (score ranging between 3 and 8.7), middle (8.8–16.9) or high (17.0–21).
